# Identification of a bacteriocin-like compound from *Lactobacillus plantarum* with antimicrobial activity and effects on normal and cancerogenic human intestinal cells

**DOI:** 10.1186/s13568-019-0813-6

**Published:** 2019-06-17

**Authors:** Alessandra De Giani, Federica Bovio, Matilde Forcella, Paola Fusi, Guido Sello, Patrizia Di Gennaro

**Affiliations:** 1 Department of Biotechnology and Biosciences, University of Milano-Bicocca, Piazza Della Scienza 2, 20126 Milan, Italy; 2 Department of Chemistry, University of Milano, Via Golgi 19, 20133 Milan, Italy

**Keywords:** *Lactobacillus plantarum*, Bacteriocin-like compounds, Antimicrobial activity, Healthy cells, Anti-tumoral activity

## Abstract

In this paper, we demonstrate that the antimicrobial activity of *L. plantarum* PBS067 strain against antagonist microorganisms was mediated by the production of a bacteriocin-like compound secreted at the stationary phase of the growth. The novel bacteriocin-like compound, designed plantaricin P1053, was identified by using sorption–desorption method, butanol extraction and SEC-HPLC. The molecular mass of plantaricin P1053 was shown to be 1053 Da by ESI-MS analysis. Plantaricin P1053 exhibited a broad-spectrum antimicrobial activity against Gram-positive bacteria as *S. aureus* and Gram-negative bacteria as *E. coli*. In addition to the antimicrobial activity, the isolated bacteriocin-like compound showed effects on normal and cancerogenic epithelial intestinal cell lines through an enhancing of viability of healthy cells and a proliferation reduction of cancer cells. Moreover, in this paper we demonstrate that the isolated bacteriocin-like compound acts on healthy cells through the epidermal growth factor receptor (EGFR) pathways. In conclusion, plantaricin P1053 isolated from *L. plantarum* PBS067 strain could represent one of the first multifunctional bacteriocin-like compound acting on human epithelial intestinal cells.

## Introduction

Probiotics are recognized as live microorganisms that confer a health benefit to the host when administered in adequate amounts (FAO/WHO 2001). These bacteria exert health-promoting properties, including the effects on specific tissues, particularly on the intestine. Most of the mechanisms involve the modification of the gut microbiota because of the immune-modulating attitude on the intestinal tissues (Valeur et al. 2004) or the release of essential micronutrients in the guts, such as group B vitamins (LeBlanc et al. 2011) and the production of substances such as organic acids or bacteriocins with antagonistic activity against potential pathogens (Servin 2004). Among the antimicrobial substances produced by these bacteria, bacteriocins are interesting because of their several applications, like natural food preservatives or novel therapeutic agents to complement conventional antibiotics (Trivedi et al. 2013). The biochemical characterization can be an important goal to understand their mode of action. In fact, bacteriocins are ribosomally synthesized antimicrobial peptides or complex proteins secreted by various Gram-positive and Gram-negative bacteria (Desriac et al. 2010). Based on biochemical properties, bacteriocins have been divided into three groups: family of lantibiotics (Class I), small non-modified peptides resistant to heat and pH (Class II), larger heat-labile proteins (Class III) (Klaenhammer 1993; Porto et al. 2017). Class II is subdivided into four sub-groups. One of the four sub-groups is the Class IIA that contains bacteriocins known as “pediocin-like” peptides whose synthesis requires four genes encoding proteins regulated by a quorum sensing mechanism (Da Silva et al. 2014; Diep et al. 2009). These proteins and peptides are known for their antimicrobial properties against some potential pathogens like *Escherichia coli*, *Staphylococcus aureus* and *Bacillus* spp. (Ahmad et al. 2017; Zhao et al. 2016). Many of these bacteriocins are produced from *L. plantarum* strains. Some of them have been studied, such as *L. plantarum* JLA-9 (Zhao et al. 2016), *L. plantarum* ZJ5 (Song et al. 2014), *L. plantarum* ZJ008 (Zhu et al. 2014). The bacteriocins isolated from these strains have the peculiarity to have a very low molecular weight around 1000–3000 Da.

The mechanism of action of bacteriocins is not well understood, but in literature it is reported that they can kill target bacteria by membrane permeabilization or by binding to a specific membrane protein called “bacteriocin receptor”, where the interaction between peptide and receptor protein leads to membrane leakage and cell death (Oppegard et al. 2016). This mechanism explains both the extreme potency of many bacteriocin-like compounds and their narrow inhibition spectra. However, we still lack knowledge about the nature of the interaction between these compounds and receptors, and also the interaction between these compounds and intestinal human cells. Some authors reported that bacteriocins can exert some beneficial effects on humans, because of their interaction with the intestinal epithelia. It is reported that metabolites from probiotic cultures showed an anti-proliferative effect on human colon cancer (do Lee et al. 2008; Ma et al. 2010; You et al. 2004). Moreover, Ma et al. (2010) speculated that the activity is mediated through the inhibition of Epidermal Growth Factor Receptor (EGFR) kinase activity. Also Yan et al. (2011) described the effect of a bacteria-derived soluble protein p40 from *Lactobacillus rhamnosus* GG, which was able to prevent cytokine induced apoptosis in intestinal epithelial cell lines through the regulation of the activation of Akt. Nevertheless, probiotic effects are strain dependent and different species might have different mechanisms of action (Dimitrovski et al. 2014).

In a previous work (Presti et al. 2015), strains of *Lactobacillus* spp. and *Bifidobacterium* spp. were characterized for their probiotic properties, among which there was the antimicrobial activity. Previous results of cell-free supernatants at neutral and acidic pH from all selected probiotic strains and the direct growth inhibition of the potential pathogens on the LABs, made us speculate that bacteriocin-like compounds can be produced, especially from *Lactobacillus plantarum* strain PBS067, showing a strong antimicrobial activity.

The aim of this work was to demonstrate that the antimicrobial activity exhibited from *L. plantarum* strain PBS067 against antagonist microorganisms was mediated by a bacteriocin-like compound, produced and secreted in the medium during the growth of the strain. The purified compound was described as likely belonging to an uncommon group of bacteriocins (Class II). The novelty of the work is that plantaricin P1053 revealed to possess both an antimicrobial activity against pathogenic bacteria and an effect on host cells through an enhancement of healthy cells viability and a reduction of cancerogenic intestinal cells viability.

## Materials and methods

### Strains and culture conditions

*Lactobacillus plantarum* strain PBS067 (Presti et al. 2015) (deposited at DSMZ culture collection as DSM 24937), isolated from the feces of healthy humans, was supplied from a private collection (Principium Europe S.r.l., now Roelmi Hpc). *L. plantarum* PBS067 was selected for this study for its ability to exhibit a strong antimicrobial activity against different human pathogens as *E. coli*, *P. aeruginosa*, *S. aureus*, *E. faecium* after 24 h of incubation at 37 °C. Unless otherwise specified, *L. plantarum* was cultured in deMan, Rogosa and Sharpe (MRS) medium. The cultures were incubated at 37 °C under anaerobic conditions using anaerobic atmosphere generation bags (Anaerogen, Oxoid).

As antagonistic microorganisms for antimicrobial activity assay, *E. coli* ATCC 25922, *Staphylococcus aureus* ATCC 6538, *Pseudomonas aeruginosa* ATCC 9027, and *Enterococcus faecalis* ATCC 2922 were employed. The cultures were performed in Luria–Bertani medium (LB) agar, modified by Lennox known as LD (Lennox 1995), at 37 °C in aerobiosis.

### Screening for antimicrobial activity

The antimicrobial activity was determined by the well diffusion agar assay (WDAA) for lactobacilli according to the protocol of Santini et al. (2010) with some modifications. Overnight MRS cultures were centrifuged at 7000 rpm at 4 °C for 15 min. The pHs of the supernatants were measured and recorded. The supernatants that were naturally at pH 4 were collected and filtered through 0.22-µm pore filter membranes to remove any residual bacterial cell (CFS, Cell Free Supernatant). The CFS antimicrobial activity was measured against *E. coli, P. aeruginosa, E. faecalis*, *S. aureus* bacteria. In particular, the antagonist strains were inoculated into LD medium and let to grow until the optical density at 600 nm (OD_600_) was 0.5, corresponding to around 10^7^ CFU/mL. 2.5% (v/v) of the culture was inoculated into 20 mL of LD agar and the plates were allowed to solidify. Four wells of 8 mm in diameter were made on each agar plate with a sterile glass cylinder. 50 µL and 100 µL of *L. plantarum* PBS067 culture supernatant (CFS) was dispensed into each well; neutralized-acidified PBS067 CFS, not-inoculated and acid not-inoculated MRS (100 µL) were used as controls. Plates were incubated overnight at 37 °C in aerobiosis. The growth inhibition haloes were measured.

The same method was used to test the antimicrobial activity of the concentrated crude extract and the purified bacteriocin-like compound against *E. coli*, and *S. aureus* as representative strains of Gram-negative and Gram-positive bacteria, respectively.

### Purification of the bacteriocin-like compound from culture medium of *L. plantarum*

#### Adsorption/desorption method (step 1)

A modification of Yang et al. (1992) extraction method, based on the hydrophobicity and the charge of the compounds secreted by the cells, was used in order to identify bacteriocin-like compounds from *L. plantarum* strain PBS067 cultures.

First, the strain was pre-cultured in 15 mL of MRS medium at 37 °C overnight, in anaerobic conditions. Then, the pre-culture was inoculated in four flasks each containing 300 mL of MRS medium with an initial 0.01 OD_600_. The flasks were incubated at 37 °C, for 16 h, in microaerophilic conditions. Final cultures had an OD_600_ between 3 and 4, which corresponded to the initial latency period.

The established method allowed to partially purify cell secreted compounds on the basis of the pH of extraction medium promoting the adsorption/desorption of these molecules from the lactic acid bacteria membrane. Therefore, in the step 1 the cultures were neutralized to pH 6.5 with NaOH 1.5 M and let to stand for 30 min at room temperature. Then, they were centrifuged at 7000 rpm at 4 °C for 10 min. After the cells had been washed with a phosphate buffer (0.1 M pH 6.5), they were resuspended in 60 mL of NaCl 0.1 M at pH 2 and mixed with a magnetic stirrer for 1 h at 4 °C. Cell suspension was then centrifuged at 16,000*g* for 30 min at 4 °C. This fraction of the supernatant containing the bacteriocin-like molecule (called crude extract) was used for further analysis and characterization.

An aliquot of this fraction containing the bacteriocin-like molecule was tenfold concentrated and used for WDAA (see above) in order to assess its antimicrobial activity using 100 µL of the concentrated crude extract. 100 µL of cell free supernatant (CFS) (see above) and 100 µL of NaCl 0.1 M at pH 2 were used as positive and negative controls, respectively. The growth inhibition zones around the wells were measured.

Before proceeding with the purification, the pH of the fraction was increased up to 10–11 with NaOH 1.5 M, and then the solution was lyophilized.

#### Organic phase extraction (step 2)

The lyophilized crude extract (deriving from 300 mL of supernatant) was extracted with 10 mL of *n*-butanol under agitation, at room temperature for 20 h. This step was repeated twice. Then, the butanol extracts were filtered through a paper filter and the solvent was evaporated. The residue was resuspended in 1 mL of methanol and analysed in SEC-HPLC.

#### Size exclusion chromatography (SEC) HPLC (step 3)

SEC analyses were performed with a Waters 600E delivery system equipped with Waters 486 UV–Vis detector and a Phenogel 5 100 A, Phenomenex, 300 × 4.6 mm column eluted in isocratic conditions with methanol at a flow rate of 0.3 mL/min. The eluted compounds were detected at 254 nm and their retention times were compared with the retention time of the standard nisin, the model bacteriocin from *Enterococcus lactis*. In these conditions, the retention time of nisin was 11.8 min. An aliquot of the eluted compound containing the bacteriocin-like molecule was tenfold concentrated and used for WDAA in order to assess its antimicrobial activity.

### Characterization of the bacteriocin-like plantaricin P1053 by ESI-full mass spectrometry (MS)

The eluted compound containing the bacteriocin-like molecule was analysed by the electrospray ionization ESI-full mass spectrometry. The analysis was performed on LCQ Fleet ion trap mass spectrometer. The MS was operated in positive ionization mode acquiring spectra in the *m/z* range of 200 to 2000.

### Effects of proteinase K enzyme on the stability of the purified bacteriocin-like compound and total protein concentration

In order to evaluate the purified bacteriocin-like molecule sensitivity to the proteinase K (pH 7.5; Sigma Aldrich, Italy), the bacteriocin-like compound was treated with 1 mg/mL (final concentration) proteinase K (ratio 1:5) at its optimal pH. After 3 h of incubation at 37 °C, the reaction was stopped at 4 °C. Subsequently, the sample was adjusted to pH 2 using 6 M HCl and assayed for antimicrobial activity. The bacteriocin-like molecule at the original pH (pH 2) without any heat or enzyme treatments was used as the control sample. The agar well diffusion assay was carried out to test the remaining activity against the indicator strain *E*. *coli* ATCC25922 (Santini et al. 2010).

The total protein concentration was assessed on the partial purified and purified bacteriocin-like molecule extracts using a Pierce BCA protein assay kit (Thermo Fisher Scientific, Italy) according to the manufacturer’s protocol. Protein concentrations were calculated from the standard curve by 20 mg/mL bovine albumin serum.

### Maintenance and growth of cell lines for in vitro tests

CCD 841 (ATCC CRL-1790) healthy colon cell line (Thompson et al. 1985) were grown in EMEM medium supplemented with heat-inactivated 10% FBS, 2 mM l-glutamine, 1% non-essential amino acids, 100 U/mL penicillin and 100 µg/mL streptomycin and maintained at 37 °C in a humidified 5% CO_2_ incubator.

E705 colon cancer cell line (supplied by IRCCS Foundation, Cancer National Institute) (Mozzi et al. 2015) was grown in DMEM medium supplemented with heat-inactivated 10% FBS, 2 mM l-glutamine, 100 U/mL penicillin and 100 µg/mL streptomycin and maintained at 37 °C in a humidified 5% CO_2_ incubator.

All the reagents for cell culture were supplied by EuroClone (EuroClone S.p.A, Pero (MI), Italy).

### Viability assay

Cell viability was assessed using an in vitro MTT based toxicology assay kit (Sigma). Cells were seeded in a 96-well micro titer plates at a density of 8 × 10^4^ cells/100 µL and incubated overnight. The attached cells were treated with a range of plantaricin P1053 up to 1 µg/mL. After 48 h of treatment, MTT test was performed according to the manufacturer’s protocol and absorption was measured at 570 nm using a micro plate reader to assay the effect of plantaricin P1053 on healthy CCD 841 cells and colon cancer E705 cells. Results were expressed as mean values ± SD of three determinations.

### Protein extraction from treated cell lines and analysis

After treatment with plantaricin P1053 at 0 min, 30 min, 1 h, 3 h and 24 h, cells were washed with ice-cold PBS and lysed in RIPA buffer, containing protease and phosphatase inhibitors and 1 mM PMSF. After lysis on ice, homogenates were obtained by passing crude extracts five times through a blunt 20-gauge needle fitted on a syringe and subsequently centrifuging them at 14,000 rpm for 30 min at 4 °C. Supernatants were analyzed for protein content by the BCA protein assay (Smith et al. 1985).

SDS-Page and western blot were carried out by standard procedures. Thirty micrograms of proteins were separated on 10% acryl-amide/bis-acrylamide SDS-PAGE, transferred onto a nitrocellulose membrane (Millipore, Billerica, MA), probed with the appropriate antibodies and visualized using ECL detection system (Millipore). Protein levels were quantified by densitometry of immunoblots using ScionImage software (Scion Corp., Fredrick, MD). We used the following primary antibodies (all purchased by Cell signaling Technology, Danvers, MA): anti-EGFR (dilution 1:1000), anti-phospho-EGFR (Tyr 1068; dilution 1:1000), anti-p44/42 MAPK (Erk1/2; dilution 1:1000), anti-phospho-p44/42 MAPK (Erk1/2) (Thr202/Tyr204; dilution 1:1000), anti-Akt (dilution 1:1000), anti-phosho-Akt (Ser473; dilution 1:1000) and anti-GAPDH (dilution 1:10,000). IgG HRP-conjugated secondary antibodies (purchased by Cell Signaling Technology) were diluted 1:10,000.

### Statistical analysis

Experiments were performed in triplicate and results were presented as mean values ± standard deviation. The statistical relevance was assessed by Student’s t test. The significance was defined as p value < 0.05.

## Results

### Identification of antimicrobial activity of *L. plantarum* strain PBS067

*Lactobacillus plantarum* strain PBS067 was grown until reaching the stationary phase of the cells and the cell free supernatant (CFS) was used in the antimicrobial activity through the well diffusion agar assay. A halo of growth inhibition of the antagonist bacteria was observed and measured (Table 1). Results showed a halo of inhibition with a diameter of 18 mm ± 0.2 for *E. coli* and 20 mm ± 0.2 for *S. aureus* as representative Gram-negative and Gram-positive bacteria, respectively.

**Table 1:** Dimension of the growth halo inhibition measured by CFS from *L. plantarum* PBS067 against the antagonist bacteria

Antagonist bacteria	Halo inhibition by *L. plantarum* PBS067 (mm)	
	50 µL^a^	100 µL^a^
*Escherichia coli* ATCC 25922	18 ± 0.2	35 ± 0.2
*Enterococcus faecalis* ATCC 2922	10 ± 0.5	20 ± 0.5
*Pseudomonas aeruginosa* ATCC 9027	20 ± 0.5	40 ± 0.5
*Staphylococcus aureus* ATCC 6538	20 ± 0.2	40 ± 0.2

The measures of the inhibition zone are expressed in mm

^a^The concentration of the solution containing the plantaricin P1053 was of 200 µg/mL

**Fig. 1:**
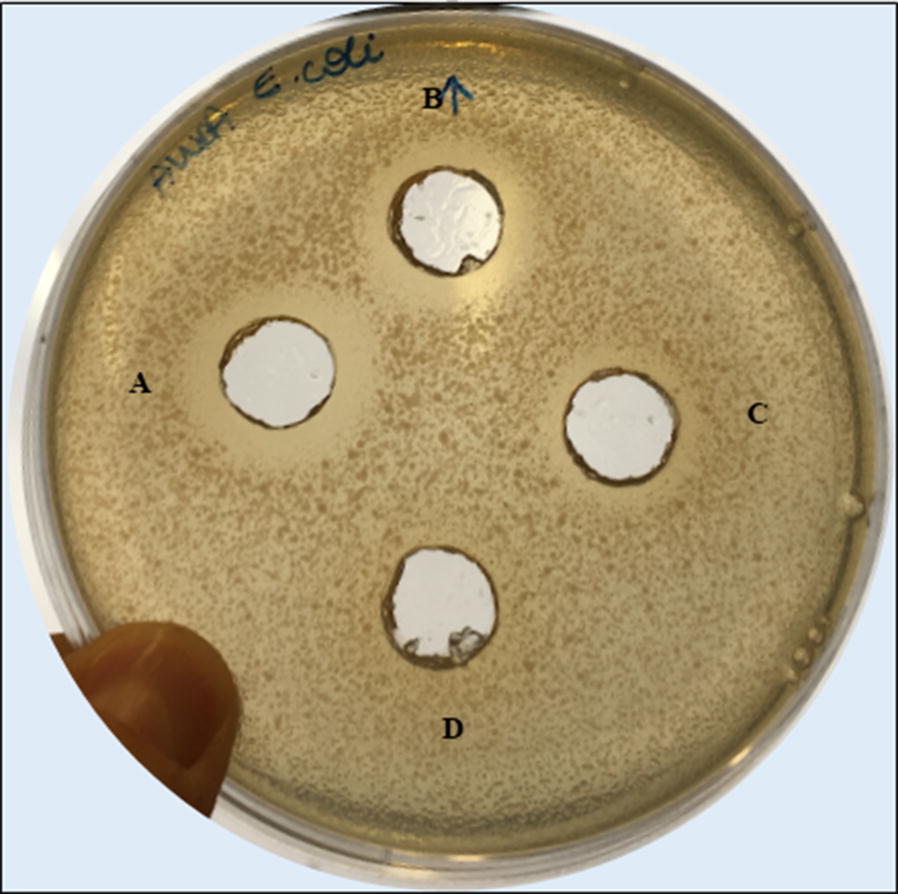
Antimicrobial activity of *Lactobacillus plantarum* PBS067 cell free supernatant. The antimicrobial activity of *L. plantarum* PBS607 cell free supernatant (CFS) was determined as halo of growth inhibition of *E. coli* ATCC 25922 through the agar well diffusion assay. The figure shows the inhibition zones produced by CFS (A), by neutralized-acidified CFS (B), not inoculated MRS at pH 4 (C), not inoculated MRS (D) against *E. coli* ATCC 25922

Results were compared to the acidified non-inoculated medium as negative control, in order to exclude that the inhibition was caused by the low pH of the medium after the growth. Moreover, obtained data showed that the antimicrobial activity was also maintained when the supernatant was neutralized and then brought again to acid pH, as reported as an example for *E. coli* in Fig. 1. For this reason, we hypothesized that the strain PBS067 was able to produce bioactive bacteriocin-like compounds inhibiting the growth of the tested antagonist bacteria. As the bacteriocin-like compounds produced by lactobacilli are strain-specific and with a peculiar kind of biological activity, we decided to isolate and characterize the bioactive compounds produced by *L. plantarum* PBS067 strain and to test their range of antimicrobial activity.

### Purification of the bacteriocin-like compound from *L. plantarum* strain PBS067

**Fig. 2:**
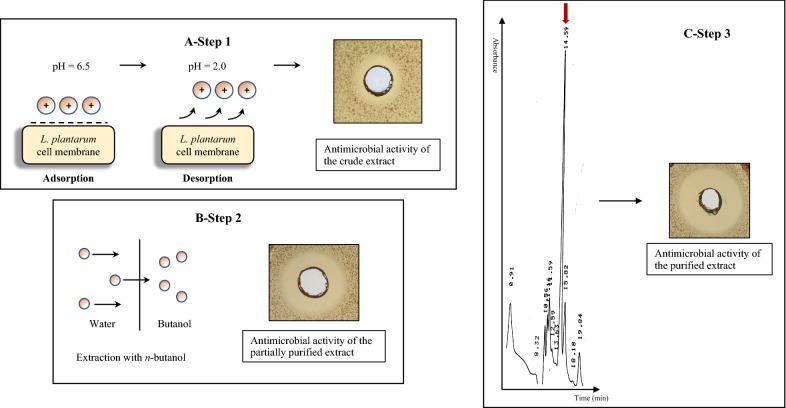
Step-by-step purification and antimicrobial activity of the bacteriocin-like compound. The antimicrobial activity of the purified extract was verified through agar well diffusion assay. Step 1 of purification and inhibition halo produced by the crude extract obtained after adsorption/desorption method (A), step 2 of purification and antimicrobial activity of the partial purified extract through an extraction with *n*-butyl alcohol (B), step 3 of purification and the growth inhibition of the eluted bacteriocin-like compound (C). All samples were tested against *E. coli* ATCC 25922

Bacteriocin-like compounds were isolated from cell free supernatant (CFS) of *L. plantarum* PBS067 culture using the modified method described by Yang et al. (1992) based on the hydrophobicity and the charge of the compounds secreted by cells. The established method allowed to extract and partially purify the bacteriocin-like molecules in a fraction called crude extract. The crude extract was screened for the antimicrobial activity using the agar well diffusion assay to verify that this property was maintained after this extraction phase (Fig. 2A, step 1). Results were compared to NaCl 0.1 M at pH 2 as negative control and CFS as positive control. The assay showed an increase in specific antimicrobial activity in this fraction (crude extract) measured through the dimension of the inhibition halo. The activity was not shown at neutral and alkaline pH, but it was recovered when the pH was lowered to 4, the lactobacilli physiological pH.

Then, the lyophilized crude extract from *L. plantarum* supernatant was submitted to another step of purification performed with *n*-butyl alcohol. After the antimicrobial activity of the partially purified extract deriving from this second step was demonstrated, the obtained extract was analyzed and characterized (Fig. 2B, step 2).

The extract deriving from *n*-butanol extraction was first analyzed in HPLC by Size Exclusion Chromatography (SEC). The UV/visible absorption spectra of the sample revealed a maximum absorbance–wavelength at 254 nm. Results from SEC-HPLC analysis showed a main peak at 254 nm with a retention time of 14.5 min whose eluate maintained the antimicrobial activity (Fig. 2C, step 3). As a preliminary characterization, the purified extract was compared with the only reference standard available, the 3354.0 Da nisin from *Lactococcus lactis*. Based on the comparison of the retention times, the main peak of the extract had a molecular weight lower than 3354.0 Da. On this basis, the ESI mass spectrometry analysis of the purified extract will be used to thoroughly characterize the bacteriocin-like compound and determine the molecular weight.

### Effects of proteinase K on the stability of the purified bacteriocin-like compound and total protein concentration

**Fig. 3:**
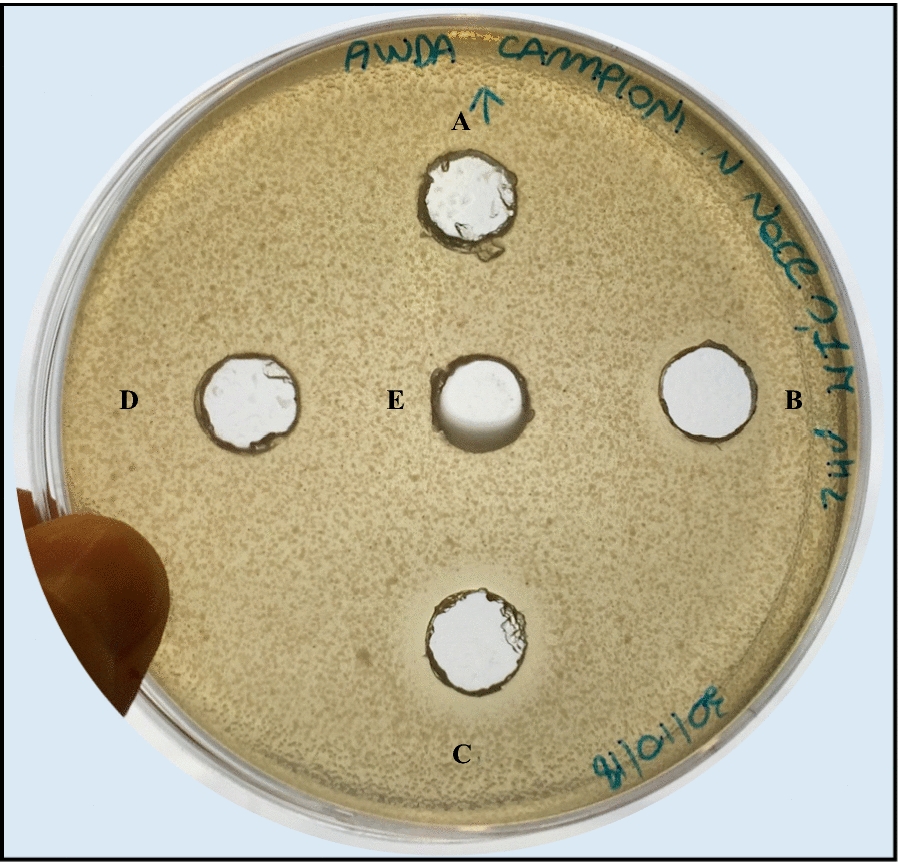
Effect of proteinase K on the purified bacteriocin-like compound. The antimicrobial activity of the purified bacteriocin-like compound was determined as halo of growth inhibition of *E. coli* ATCC 25922 through the agar well diffusion assay. The Figure shows the lack of inhibition zone produced by the treatment of the bacteriocin-like compound by proteinase K (A); the inhibition halo produced by the no-treated bacteriocin-like compound after incubation at 37 °C and pH 7.5 (the proteinase K optimal pH) (B); the inhibition zone produced by the untreated bacteriocin-like compound at pH 2 (its optimal pH), as a positive control of the test (C); the lack of inhibition halo produced by the negative control NaCl 0.1 M pH 7.5 (buffer of the reaction with proteinase K) (D); and the lack of inhibition halo produced by the negative control NaCl 0.1M pH 2 (buffer for bacteriocin-like molecule activity) (E)

The sensitivity of the purified bacteriocin-like molecule to the proteinase K was evaluated after 3 h of incubation at 37 °C with the hydrolytic enzyme. Results indicated that the treatment with proteinase K showed the expected effect on the purified bacteriocin-like molecule; in fact, the inhibitory activity of the purified bacteriocin-like molecule against *E*. *coli* strain ATCC25922 was completely lost (Fig. 3) after the treatment.

The crude extract and the purified bacteriocin-like molecule were positive to BCA protein assay. In particular, the total protein concentration of the crude extract was 200 µg/mL, while the corresponding expected concentration was 3900 µg/mL based on the initial dry weight of the supernatant. After the purification of the bacteriocin-like molecule, the total protein concentration was 4000 µg/mL, while the corresponding calculated concentration was 4140 µg/mL based on the lyophilized amount. Results demonstrated that the bacteriocin-like compound was a protein-like molecule and that after purification a 100% enrichment of proteins was determined.

### Characterization of the bacteriocin-like compound by ESI mass spectrometry

**Fig. 4:**
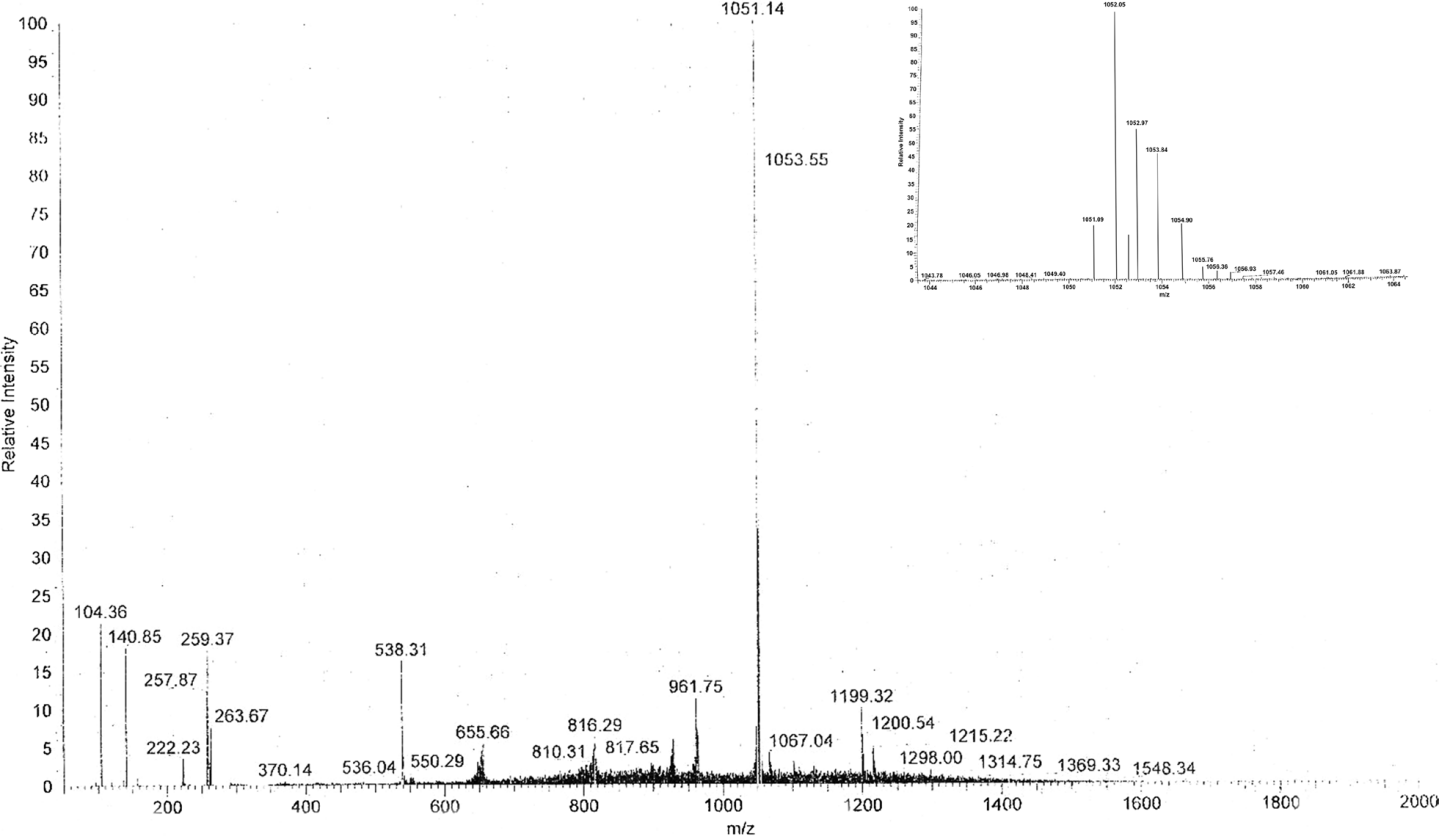
Analysis of the bacteriocin-like compound by mass spectrometry. Full scan mass spectrum of the extract in positive ESI ion detection mode with one main fragment showing *m/z* = 1053.05 Da. ITMS + c HESI E full MS (1.16 e4). The area around the M+ is enlarged in the insert

We decided to perform an ESI-full MS analysis on the enriched fraction of our bacteriocin-like compound. This analysis revealed that the extract contained a main compound with a molecular weight of 1053 Da (Fig. 4) (from here named plantaricin P1053); smaller than the most important plantaricins described in literature as for example plantaricin C19 (3.8 kDa) from *L. plantarum* C19 (Atrih et al. 2001), but with a molecular weight similar to those of JLA-9 from *L. plantarum* JLA-9 (Zhao et al. 2016). The weight is in agreement with the results obtained by SEC-HPLC chromatography.

### Antimicrobial spectrum activity of plantaricin P1053

**Fig. 5:**
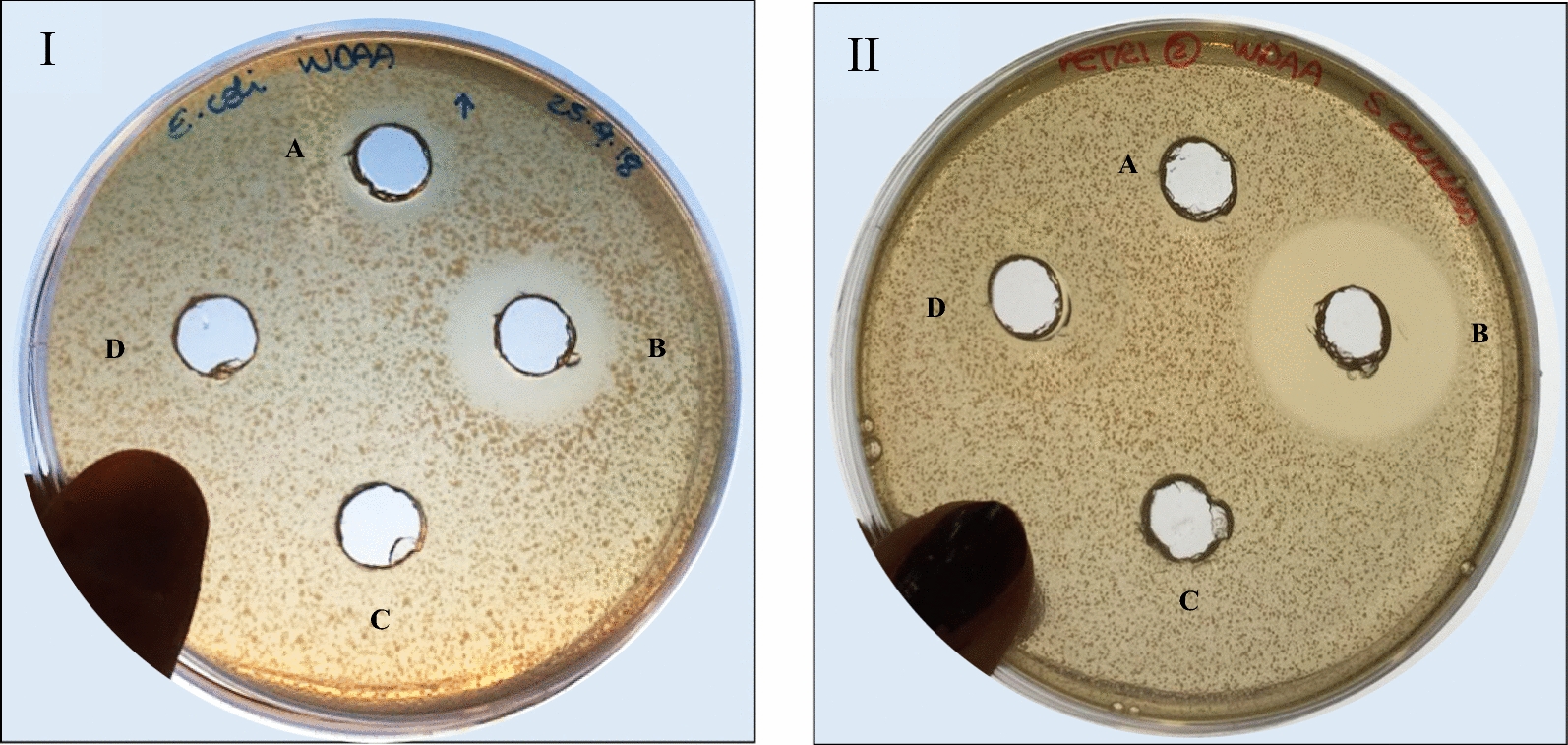
Antimicrobial spectrum activity of plantaricin P1053. The antimicrobial spectrum activity of the plantaricin P1053 was verified through the inhibition growth halo in the agar well diffusion assay. The first panel shows the inhibition haloes against *E. coli* ATCC 25922 and the second panel against *S. aureus* ATCC 6538 produced by the plantaricin P1053 at neutral pH (A), by the plantaricin P1053 at pH 2 (B), by the NaCl 0.1 M at pH 2 (C) and by NaCl 0.1 M at pH 6.5 (D) as negative controls

Antimicrobial spectrum activity of the purified plantaricin P1053 was determined by the measure of the halo inhibition of the growth against *E. coli* and *S. aureus* as representative of Gram− and Gram+ antagonist bacteria, respectively. Results are showed in Fig. 5. Plantaricin P1053 exhibited a notable antimicrobial activity against both *E. coli* and *S. aureus* with a growth inhibition zone of 2.5 cm and 3.0 cm, respectively. The inhibition spectrum of plantaricin P1053 appeared to be relatively wide because of the activity against both Gram-negative and Gram-positive bacteria.

In addition, the minimum inhibition growth concentration (MIC) of plantaricin P1053 against these bacteria was determined and data are reported in Table 2. Results show that the MIC values are 7.8 µg/mL for *E. coli* strain ATCC25922 and 31 µg/mL for *S. aureus* strain ATCC 6538, respectively.

**Table 2:** Minimum inhibitory concentration (MIC) of the growth measured by plantaricin P1053 isolated from *L. plantarum* strain PBS067 against *E. coli* ATCC 25922 and *S. aureus* ATCC 6538

Plantaricin P1053 (μg/mL)	*E. coli* ATCC 25922 growth inhibition (%)	*S. aureus* ATCC 6538 growth inhibition (%)
250.00	65.74	89.55
125.00	62.70	91.29
62.50	64.12	96.02
31.25	62.95	69.25
15.62	66.25	0.00
7.81	68.85	0.00
3.90	14.41	0.00

These data indicated an antimicrobial activity similar to those of the plantaricin JLA-9, which presented a MIC of 16 µg/mL against *S. aureus* and 64 µg/mL against *E. coli* strain ATCC25922.

### In vitro test of plantaricin P1053 on healthy colon CCD 841 cell line

**Fig. 6:**
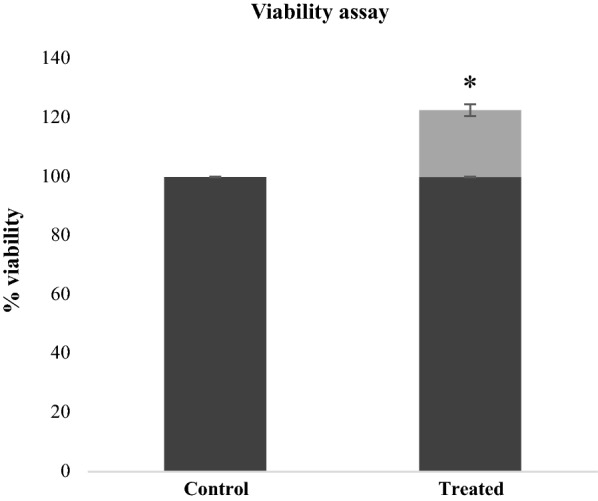
Evaluation of cell viability on normal CCD 841 cell line after treatment with plantaricin P1053. MTT test was performed on normal CCD 841 cell line. Buffer NaCl 0.1 M pH 2 was used as control and it did not have any effect on cell viability. Values are presented as mean ± SD. Statistical analyses were performed using the Student’s t-test. *p < 0.05

In order to verify that plantaricin P1053 could have beneficial effects also on human intestinal epithelial cells, we initially verified the viability of the healthy colon CCD 841 cell line in the presence of the obtained plantaricin. To this end, the viability of CCD 841 cells treated with 1 µg/mL of plantaricin P1053 was assayed by MTT test. Results reported in Fig. 6 show an increase in CCD 841 cell viability of about 20%. It appears that plantaricin P1053 acts on healthy cells as previously demonstrated for a few bacteriocins isolated from lactobacilli (Wang et al. 2014; Tao et al. 2005). So, we decided to investigate whether the isolated bacteriocin-like molecule was able to influence some pathways in healthy human cell lines.

To evaluate a possible role for plantaricin P1053 in the complex pathway triggered by EGFR activation in healthy cell lines, cells were treated with 1 µg/mL of plantaricin P1053 for different times (0 min, 30 min, 1 h, 3 h and 24 h). We decided to use CCD 841 cells because they represent the wild type cell line (healthy cells) for all the proteins involved in EGFR pathway (Akt and Erk proteins are not mutated).

**Fig. 7:**
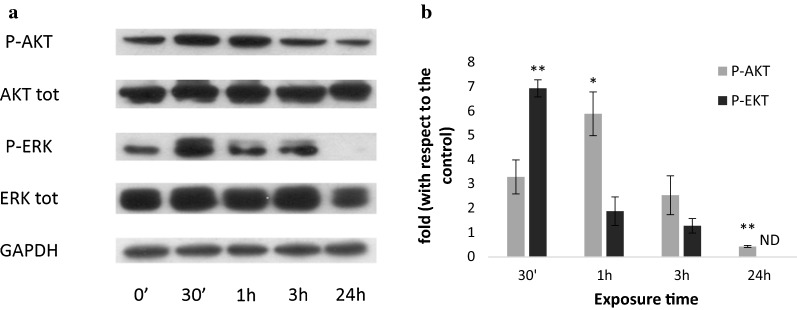
Western blotting analysis of EGFR pathway activation after exposure to plantaricin P1053 in CCD 841 cell line. Representative western blotting analyses performed on crude extracts, using anti-P-AKT, anti-AKT, anti-P-ERK1/2, anti-ERK1/2 and anti-GAPDH antibodies. GAPDH was used as a control. The experiments were performed in triplicate (**a**). Densitometric analysis performed with Scion Image Software. Values are expressed by comparing the data obtained after the treatment with plantaricin P1053 with those obtained from 0’ of exposition to the compound (**b**). Values are presented as mean ± SD. ND, no protein phosphorylation was detected

Western blot analysis showed an activation of EGFR pathway in the healthy human cell line CCD 841. Plantaricin P1053 administration to CCD 841 cells led to an increase of phospho-Akt as an early response (30 min–3 h) and a significant decrease as a late response (24 h). Erk showed a peak in its phosphorylation level after 30 min of exposure to the bacteriocin-like compound, that decreased gradually from 1 to 3 h after the administration, becoming completely unphosphorylated (ND) after 24 h (Fig. 7).

### In vitro test of plantaricin P1053 on colon cancer E705 cell line

**Fig. 8:**
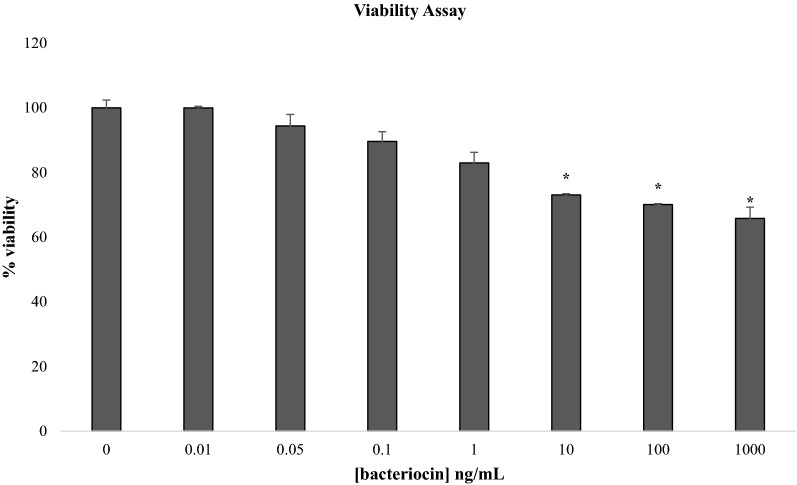
Evaluation of cell viability on E705 cancer colon cell line after treatment with plantaricin P1053. MTT test was performed on cancerogenic E705 cell line. Buffer NaCl 0.1 M pH 2 was used as control and it did not have any effect on cell viability (100% viability). Values are presented as mean ± SD. Statistical analyses were performed using the Student’s t-test. *p < 0.05

A possible beneficial effect of plantaricin P1053 on cancerogenic epithelial intestinal cells was also evaluated. A viability test of E705 cells treated with different concentrations of plantaricin P1053 was performed. Results showed a significant inhibitory effect, near 30%, on E705 cells proliferation in a concentration dependent manner. The higher concentration of plantaricin 1053 (1 µg/mL) evidenced the higher inhibitory effect, as reported in Fig. 8.

Also in this case a possible role for plantaricin P1053 in the complex pathway triggered by EGFR activation in cancerogenic cell lines, was evaluated. For this, cells were treated in the same manner with plantaricin P1053 for different times (0 min, 30 min, 1 h, 3 h and 24 h). By Western blot analysis no phosphorylation of Akt or Erk was detected, although both proteins were detected in their unphosphorylated forms (data not shown).

## Discussion

This work describes the isolation and characterization of a bacteriocin-like compound produced by *Lactobacillus plantarum* strain PBS067 that shows an antimicrobial activity against potential human pathogens and that affects both normal and cancerogenic human intestinal cells. The probiotic strain, isolated from healthy patients, was able to produce the maximum amount of a bacteriocin-like compound at the onset of the stationary phase of the growth, during an incubation for 16 h at 37 °C at pH 4. This result is quite similar to that showed by Messi et al. (2001), who reported that *L. plantarum* 35d started to produce bacteriocin during the late logarithmic growth phase. Plantaricin 35d reached the maximum concentration after 19 h of incubation (stationary phase), at pH 4.

Preliminary experiments evidenced that the cell free supernatant (CFS) of *Lactobacillus plantarum* PBS067 culture had an inhibition of the antagonist bacteria growth, as *E. coli* and *S. aureus*. Thus, a purification procedure of the CFS metabolites was performed.

On the basis of the pH dependence, we decided to use the adsorption/desorption method as the first step in the purification procedure (Yang et al. 1992). The second step of purification consisted of the extraction with a solvent like *n*-butanol, and the last consisted of the elution in SEC-HPLC. At each purification step, the antibacterial activity was tested and a comparison of the activity of the acidified purified compound in respect to the crude CFS, was verified. Results showed an increase of the antimicrobial activity along the purification procedure.

The purified compound from supernatant of *L. plantarum* strain PBS067 was then characterized. An enzymatic hydrolytic activity by proteinase K was demonstrated on the isolated compound.

The mass analysis by ESI-full MS determined a molecular mass of 1053 Da for the compound (plantaricin P1053). Because of its bactericidal activity, its behavior based on the hydrophobicity and the charge of the bioactive compound secreted by the cells, pH resistance and low molecular mass, plantaricin P1053 could be classified as a small bacteriocin-like presumably belonging to Class II, according to the definition given by Klaenhammer (1993).

Our data are in line with the results obtained by Zhao et al., where for the first time a plantaricin with a low molecular weight of 1044 Da able to act against *Bacillus* spp. was isolated from *L. plantarum* strain JLA-9 (Zhao et al. 2016) and obtained by Zhu et al. that described the identification of the plantaricin ZJ008 with a MW of 1335 Da able to act against Gram-positive and Gram-negative bacteria (Zhu et al. 2014).

Interestingly, this paper demonstrates not only that the bacteriocin-like compound isolated from *L. plantarum* PBS067 showed a broad range of antimicrobial activity towards Gram-positive and Gram-negative bacteria with a good MIC, but also its activity towards human cells, extending its role from a bacteriocin to a multifunctional factor that has effect both on normal and cancer cells.

In fact, in this work we investigated also plantaricin P1053 effect towards human cells using both healthy and cancerogenic cell lines. Plantaricin P1053 was tested on human intestinal epithelial cells. Unlike other authors, like Dimitrovski et al. (2014) who used the supernatant of the broth culture, in this study we administered only the bacteriocin-like molecule resuspended in NaCl buffer. The plantaricin P1053 increased the vitality of healthy CCD 841 cell line. These results made us speculate that this bacteriocin-like compound could act on pathways involved in cell survival and proliferation, such as the epidermal growth factor receptor (EGFR) pathway. We have decided to investigate this pathway because it has been reported by Wang et al. (2014) that a *Lactobacillus rhamnosus* GG-derived soluble protein, p40, is able to activate EGFR and its downstream target Akt in intestinal epithelial cells, leading to an inhibition of apoptosis and a preservation of the barrier function by an upregulation of mucin production. Moreover, Tao et al. (2005) reported that a soluble factor from the probiotic strain *Lactobacillus rhamnosus* GG could rapidly activate the MAPKs. The activation of EGFR, Akt and Erk was therefore investigated after plantaricin P1053 administration at different times. This compound is able to rapidly activate, within 30 min, Akt and Erk in healthy intestinal cells. The phosphorylation of Akt leads to an anti-apoptotic effect, while Erk activation is pro-proliferative. It is important to note that Akt and Erk activation is finely controlled, with a decrease in their phosphorylation level till a nearly switch off 24 h after treatment.

Concerning the effect of plantaricin P1053 on cancerogenic epithelial cell lines, we observed a significant loss of viability, near the 30%, of E705 cells in a concentration dependent manner. These data are in line with those of Dimitrovski et al. on different cancerogenic cell lines (Dimitrovski et al. 2014), although they used only the supernatant obtained from *L. plantarum* cultures.

SDS-PAGE electrophoresis showed no Akt and Erk activation in cancerogenic E705 cells, leading us to think that the difference in the vitality between healthy and cancer cells is due to EGFR downstream targets activation in the former. Moreover, our experiments show that plantaricin P1053 is able to reduce E705 cancer cells viability, although the mechanism involved is still not elucidated; at the same time its effect on healthy intestinal cells is an increase in viability, due to a transient phosphorylation of both Akt and Erk which is achieved with a different timing and is readily switched off in 24 h.

In conclusion, in this paper we have demonstrated that the antimicrobial activity of *L. plantarum* PBS067 is mediated by the plantaricin P1053 action. The novelty of the paper is that in addition to exhibiting an antimicrobial activity against both representative Gram-positive and Gram-negative bacteria, this compound can also affect the host cells through an enhancing of healthy cells and a reduction of cancer cells viability. Although some molecular mechanisms must be elucidated in further studies, plantaricin P1053 could represent one of the first multifunctional bacteriocin-like compound on human epithelial intestinal cells.

## Data Availability

All data generated during this study are included in this article and its additional files.
